# Surface-Wave Based Model for Estimation of Discontinuity Depth in Concrete

**DOI:** 10.3390/s18092793

**Published:** 2018-08-24

**Authors:** Eunjong Ahn, Hyunjun Kim, Sung-Han Sim, Sung Woo Shin, John S. Popovics, Myoungsu Shin

**Affiliations:** 1 School of Urban and Environmental Engineering, Ulsan National Institute of Science and Technology (UNIST), Ulsan 44919, Korea; eunjong@unist.ac.kr (E.A.); guswns3@unist.ac.kr (H.K.); ssim@unist.ac.kr (S.-H.S.); 2 Department of Safety Engineering, Pukyong National University, Pusan 48513, Korea; shinsw@pknu.ac.kr; 3 Department of Civil and Environmental Engineering, University of Illinois at Urbana-Champaign (UIUC), Urbana, IL 61801, USA; johnpop@illinois.edu

**Keywords:** surface-wave transmission, self-calibrating measurement, spectral energy transmission ratio, crack depth estimation model

## Abstract

In this paper, we propose an accurate and practical model for the estimation of surface-breaking discontinuity (i.e., crack) depth in concrete through quantitative characterization of surface-wave transmission across the discontinuity. The effects of three different mixture types (mortar, normal strength concrete, and high strength concrete) and four different simulated crack depths on surface-wave transmission were examined through experiments carried out on lab-scale concrete specimens. The crack depth estimation model is based on a surface-wave spectral energy approach that is capable of taking into account a wide range of wave frequencies. The accuracy of the proposed crack depth estimation model is validated by root mean square error analysis of data from repeated spectral energy transmission ratio measurements for each specimen.

## 1. Motivations and Objectives

The presence of cracks in concrete structures can cause serious safety and/or durability problems, which may result in fatal disasters requiring tremendous social (life and monetary) costs [1,2]. Hence, it is an important responsibility of civil engineering professionals to provide effective methods for detecting, assessing, and repairing cracks in concrete structures. Given these needs, nondestructive techniques that can be applied for the detection and assessment of concrete cracks in the field have been suggested by multiple researchers in recent years [3,4,5,6,7,8]. 

From a broad viewpoint, nondestructive methods developed for the evaluation of the damage condition of concrete to date include visual and optical inspections and stress-wave based methods, as well as nuclear, magnetic, and electronic methods [3]. Of these methods, ultrasonic techniques involve measurements of pulse velocity [9,10,11], characteristics of guided waves [12], surface-wave transmission [13,14,15,16,17,18,19,20,21,22,23,24], acoustic emission [5], diffuse ultrasound [25,26,27,28], coda wave interferometry [28,29], and nonlinear ultrasound [30,31]. Each of the ultrasonic techniques makes use of different characteristics of ultrasound to detect the location and propagation of cracks, to estimate the width and/or depth of cracks, or to evaluate the durability and residual mechanical properties of concrete [32]. 

The depth of a crack is generally related to the width of the crack, which can be relatively easily measured by means of optical microscopy or image scanning [33,34]. However, the precise estimation of a crack depth is a difficult challenge until now due to the inherent heterogeneity and nonlinear fracture characteristics of concrete. In the early development of ultrasonic test methods for concrete structures, the first arrival time and corresponding P-wave velocity have dominantly been used to estimate the damage conditions of concrete [9,10,11]. However, the time of flight diffraction (TOFD) method turned out to produce considerable errors and variances for the estimation of crack depth due to the heterogeneous material properties and the lack of clearly defined crack tips in concrete [16]. Recently, diffuse ultrasound techniques have been tried to estimate the depth of cracks in concrete [26,27]. However, it was reported that diffusivity coefficients measured for the same specimen could have a considerable variation due to the selected locations of sensors [25].

Over the last two decades, surface-wave based techniques have been developed to estimate the depth of cracks in concrete structures. The transmission and reflection of Rayleigh surface waves across a crack are sensitive to the crack depth as well as the wave frequency [35]. Angel and Achenbach [36] proposed a theoretical solution for the relationship between the normalized transmission ratio (transmission ratio for a crack depth divided by transmission ratio for no crack) and the normalized crack depth (crack depth divided by the wavelength) in isotropic, linear elastic, and homogeneous materials based on numerical analyses. However, considerable variations were observed between the experimental and theoretical results for concrete [14], which is reasonable since concrete is far from being homogeneous or isotropic at the wavelength scale. Song et al. [14] suggested that the surface-wave test results show a relatively good agreement with Angel and Achenbach’s theoretical solutions only when the normalized crack depth is smaller than 0.4 or larger than 1.0. In contrast, Kee and Zhu [15] argued that the theoretical model is useful when the normalized crack depth is smaller than 0.3. 

Popovics et al. [13] applied a self-calibrating method to compensate for the variability of impact sources and contact conditions between sensors and concrete surfaces. Kim et al. [18] proposed self-compensating frequency response functions to exclude the effects of noises and mounting conditions in estimating the crack depth. To relieve the contact issues between sensors and concrete surfaces, several researchers [15,19,20,22] investigated the applicability of air-coupled sensors for surface-wave tests. Shin et al. [16,17] introduced a spectral energy concept for the analysis of the transmission of surface waves. They suggested that an increase in the crack depth caused a reduction in the spectral energy transmission ratio, and the trend was much more distinct compared with the surface-wave transmission ratio itself. Recently, Kee and Zhu [19] studied the transmission of surface waves across multiple distributed cracks with various numbers, distances between cracks, and depths. In addition, Kee and Zhu [20] observed that the transmission ratio of surface waves increased when a fully opened crack evolved to a partially closed crack due to external forces. Also, Kee and Zhu [21] investigated the effects of sensor locations on the measurement of the transmission of surface waves across a crack due to near-field wave scattering. In et al. [22] tried to estimate crack depth using air-coupled transducers as both transmitter and receiver. Shin et al. [23] estimated crack depth based on transmission of surface wave combined with principal component analysis (PCA) and neural network (NN) techniques. Kee and Nam [24] tried to estimate crack depth automatically using transmission of surface wave measured through piezoelectric (PZT) sensors.

Most previous surface-wave studies focused on the improvement of the test configuration and/or the qualitative assessment of the effects of crack depth on surface-wave transmission. However, practical models to use those data for the estimation of crack depth in concrete have been lacking. In addition, the influence of concrete properties on surface-wave transmission has not been clearly understood, and the verification of self-calibrating measurements carried out on varying concrete mixtures has not been established.

Given the aforementioned concerns, this study proposes a practical approach for the accurate estimation of surface-breaking discontinuity (i.e., crack) depth in concrete using surface-wave transmission data. The surface-wave tests of this paper are conducted with three different concrete mixtures (differing the maximum aggregate size and the concrete strength) and four different simulated crack depths. The crack depth estimation model is developed based on a spectral energy approach that is capable of taking into account a wide frequency range of waves. The proposed crack depth model illustrates a good accuracy regardless of the concrete mixture type.

## 2. Theoretical Background on Surface Wave Techniques

**Figure 1:**
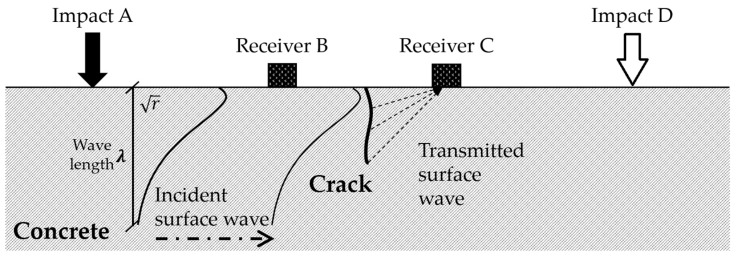
Transmission of surface waves across a crack in concrete [32].

The geometric formation (e.g., depth) of a surface crack in concrete may be characterized by the transmission behavior of surface waves across the crack. Figure 1 illustrates a typical test configuration used to examine the transmission of surface waves over a surface crack in concrete. Physical impacts (e.g., drops of a steel ball) are applied on the concrete surface near the crack to generate surface waves with relatively low frequencies, primarily lower than 50 kHz. Sensors (e.g., accelerometers, microphones) are mounted on the concrete surface at selected locations before and after the crack to measure the signal intensities of the transmitted surface waves. Impact events are applied at point A, and wave signals (e.g., accelerations normal to the surface) generated by the impacts are measured at points B (before the crack) and C (after the crack), as shown in Figure 1. The received signals at points B and C from the impact at point A in the frequency domain can be represented by:

(1) $$X_{AB}\left(f\right)=I_{A}\left(f\right)T_{AB}\left(f\right)R_{B}\left(f\right)$$

(2) $$X_{AC}\left(f\right)=I_{A}\left(f\right)T_{AB}\left(f\right)T_{BC}\left(f\right)R_{C}\left(f\right)$$

where $X_{ij}\left(f\right)$ is the Fourier-transformed signal measured at point *j* due to an impact at point *i*, $I_{i}\left(f\right)$ is the frequency response function of an impact source at point *i*, $R_{j}\left(f\right)$ is the frequency response function of a receiver at point *j*, and $T_{ij}\left(f\right)$ represents the surface-wave transmission ratio between points *i* and *j*.

Contact conditions between the sensors and concrete surfaces, as well as between the impact sources and concrete surfaces, may cause considerable variations in the surface-wave signals [13,14,16,17,18]. Therefore, to avoid the effects of different contact conditions at the sensor and impact sources, the self-calibrating method [13,14,15,16,17,18,19,20,21,22] was applied in this study. The principle of the self-calibrating method is based on the assumption that the propagation of surface waves generated by the impacts at both sides of a crack passes through the same travel path. Thus, the received signals at points C and B from the impact at point D in Figure 1 can be represented by:

(3) $$X_{DC}\left(f\right)=I_{D}\left(f\right)T_{DC}\left(f\right)R_{C}\left(f\right)$$

(4) $$X_{DB}\left(f\right)=I_{D}\left(f\right)T_{DC}\left(f\right)T_{CB}\left(f\right)R_{B}\left(f\right)$$

Here, $T_{CB}\left(f\right)$ represents the surface-wave transmission ratio between points C and B, which is assumed to be identical to $T_{BC}\left(f\right)$. Then, the transmission ratio across the crack between points B and C, $T_{BC}\left(f\right)$, can be determined by

(5) $$\left|T_{BC}\left(f\right)\right|=\sqrt{T_{BC}\left(f\right)T_{CB}\left(f\right)}=\sqrt{\frac{X_{AC}\left(f\right)X_{DB}\left(f\right)}{X_{AB}\left(f\right)X_{DC}\left(f\right)}}$$

The self-calibrating method based on the same travel path assumption is valid for cracks perpendicular to the surface, but its applicability to inclined cracks should be examined through future study.

To determine a reliable frequency domain of surface-wave measurements, the signal consistency index at each frequency, *SC*(*f*), is defined as the ratio between geometric and arithmetic averages of five independent transmission ratios [13]:

(6) $$SC\left(f\right)=\frac{\sqrt[{5}]{T_{BC1}^{d_{i}}T_{BC2}^{d_{i}}T_{BC3}^{d_{i}}T_{BC4}^{d_{i}}T_{BC5}^{d_{i}}}}{\left(T_{BC1}^{d_{i}}+T_{BC2}^{d_{i}}+T_{BC3}^{d_{i}}+T_{BC4}^{d_{i}}+T_{BC5}^{d_{i}}\right)/5}$$

Here, the five transmission ratios, $T_{BC1}^{d_{i}}$ to $T_{BC5}^{d_{i}}$, are acquired from five independent surface-wave measurements between two points, *B* and *C*, in a specimen with a crack depth of *d_i_*. 

The transmission ratio of surface waves generally decreases with an increase in the crack depth for most frequencies. However, the change in the transmission ratio was not consistent with the variation in the wave frequency, and was not always proportional to the variation in the crack depth at certain frequencies [13,16,17]. To eliminate the effect of the dependency of the transmission ratio on the frequency, Shin et al. [16,17] introduced a spectral energy concept for relating the crack depth to the transmission characteristics of surface-waves. They defined the surface-wave spectral energy by:

(7) $$SE\left(d_{i}\right)=\int_{f_{L}}^{f_{U}}\left|T_{BC}\left(f,d_{i}\right)\right|df$$

Here, $SE\left(d_{i}\right)$ is the surface-wave spectral energy for a crack depth of *d_i_*, and $f_{L}$ and $f_{U}$ are the lower and upper frequency limits used for the integration of surface-wave transmission ratios. The lower and upper integral limits in this equation can be determined based on the signal consistency index (*SC*). In addition, the spectral energy transmission ratio was defined as the spectral energy for a considered crack depth normalized by the spectral energy for no crack [16,17];

(8) $$SETR\left(d_{i}\right)=\frac{SE\left(d_{i}\right)}{SE\left(d_{0}\right)}=\frac{\int_{f_{L}}^{f_{U}}\left|T_{BC}\left(f,d_{i}\right)\right|df}{\int_{f_{L}}^{f_{U}}\left|T_{BC}\left(f,d_{0}\right)\right|df}$$

where $SETR\left(d_{i}\right)$ is the surface-wave spectral energy transmission ratio for a crack depth of *d_i_*, and $SE\left(d_{0}\right)$ is the surface-wave spectral energy for no crack. Note that the proposed method is dependent on the availability of reference-state data for a crack-free condition.

## 3. Test Description

**Figure 2:**
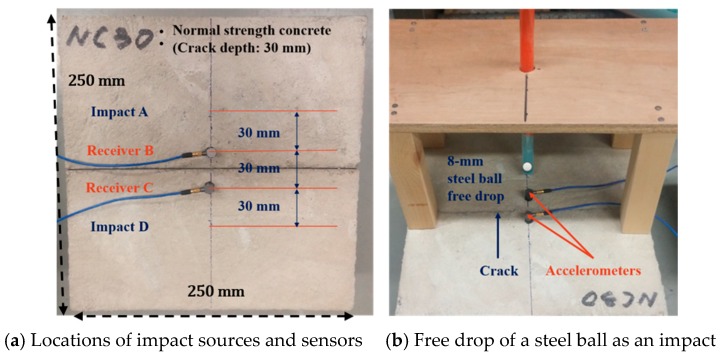
Experimental setup.

The purpose of this experimental study was to quantify the transmission characteristics of surface waves in concrete with various crack depths and mix proportions, so as to propose a practical model for the estimation of crack depth. Each specimen represented a concrete slab with a flexural crack, and was fabricated to have an artificial crack using a notch (Figure 2). The use of a notch instead of a real crack is justified by the preceding results that the transmission of surface waves was not significantly affected by the width or type of cracks (e.g., generated by notches or by three-point bending tests) [13]. Twelve specimens were prepared with four different notch depths and three different concrete mix proportions (Table 1).

### 3.1. Test Variables

The test variables of this study included the crack depth, presence of coarse aggregate, and concrete compressive strength, as shown in Table 1. In the labels of the specimens, the letter abbreviation “M,” “NC,” or “HC” signifies mortar, normal strength concrete, or high strength concrete, and the following number (i.e., 0, 15, 30, or 45) indicates the depth of the crack in mm. Four different crack depths were tested, and for each crack depth, three different mix proportions of concrete were prepared. Normal strength concrete was designed as the control case with a maximum size of a coarse aggregate of 19 mm, a water-cement ratio of 0.45, and a fine-to-total aggregate ratio of 0.5. Both mortar and normal strength concrete contained the same relative proportions of cement, water, and fine aggregate, and presented similar compressive strengths. Both high and normal strength concretes included the same amounts of coarse and fine aggregates in the unit volume of concrete, and had the same maximum size of coarse aggregate. 

The compression test results of the three mixtures in Table 1 are summarized in Table 2. Standard cylindrical specimens of 100 mm in diameter were used for the compression tests. The compressive strengths of normal and high strength concretes were approximately 40.1 and 57.4 MPa at 28 days of curing, respectively, and the strength of mortar was similar to that of normal strength concrete. Both normal and high strength concretes exhibited similar initial moduli of elasticity, but that of mortar was much lower due to the absence of coarse aggregate.

### 3.2. Specimen Fabrication

To minimize the effects of reflected wave signals from the bottom and side faces of the specimen, the specimen was designed to be sufficiently wide and deep: 250 mm in width, 250 mm in length, and 100 mm in depth (Figure 2). The dimensions of the specimen were determined based on the results of preliminary numerical simulations. A single surface-breaking was fabricated at the mid-span of each specimen using a 1 mm-wide stainless steel plate that was placed in the mold at the mid-length of the slab before casting and removed after the concrete achieved set, thus leaving behind a thin air-filled notch of well-defined depth. This notch is used to simulate a crack of the same depth, and will be referred to as a “crack” henceforth. The amount of super-plasticizer for each mixture (Table 1) was adjusted during mixing to prevent any possible flaw in the hardened specimen by providing a proper flow of the fresh mixture. The cast specimens were demolded after one day of curing. The specimens for the surface-wave tests as well as the cylindrical specimens for the compression tests were cured in water for 28 days. After curing, the surfaces of the specimens were polished using a grinder at the locations of sensors and impacts. 

### 3.3. Test Configuration

For the test setup, two impact locations were defined at points A and D, and two signal receivers were attached at points B and C, as shown in Figure 2. Two small one-dimensional accelerometers (PCB 353 B15 with flat frequency responses to 30 kHz) were used as the signal receivers to measure the accelerations normal to the specimen surfaces, and were attached 15 mm apart from the crack on each side. Data were collected using NI USB-6251. All signals from the receivers were collected using LabVIEW (National Instruments Corporation, Austin, TX, USA) and analyzed using MATLAB (Mathworks, Natick, MA, USA). 

A set of two separate impacts were applied at points A and D in a row, as shown in Figure 2a (first at 30 mm apart from receiver B and next at 30 mm apart from receiver C). To apply an impact on the surface of the specimen, a steel ball with 8 mm diameter was dropped at a height of 200 mm from the surface, as shown in Figure 2b. The diameter of the steel ball was chosen to satisfy the following two requirements. For the specimen to suitably represent a half-infinite solid medium, the wavelengths of the generated surface waves should be shorter than the thickness of the specimen (i.e., 100 mm). However, to characterize the transmission of surface waves over the cracks in the test samples, a major portion of the surface waves should have wavelengths longer than the largest crack depth (i.e., 45 mm). 

## 4. Data Processing

**Figure 3:**
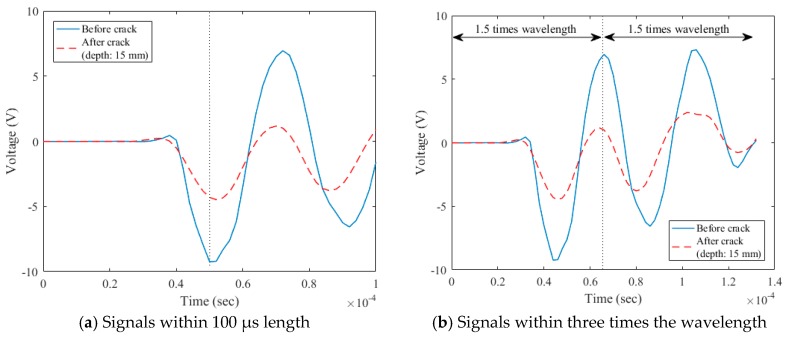
Surface-wave components extracted from raw time-domain signals.

**Figure 4:**
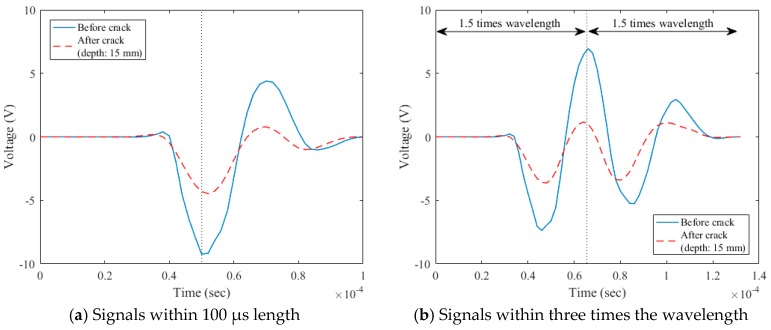
Time-domain signals windowed by Hann function.

**Figure 5:**
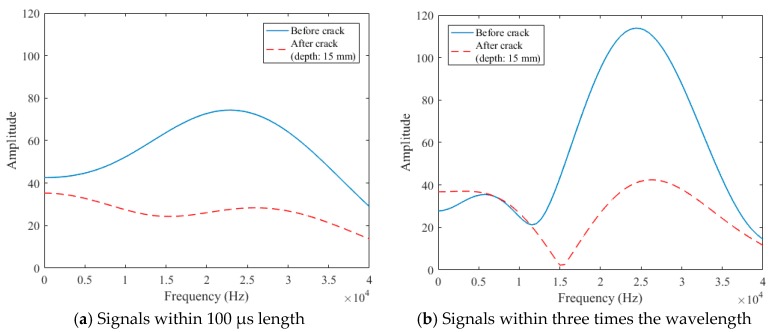
Frequency-domain signals transformed by fast Fourier transform (FFT) function.

The sampling rate of the data acquisition (DAQ) used to measure surface wave signals was 500 kHz. The data processing procedures are described in Figure 3, Figure 4 and Figure 5. As a useful time domain to analyze the transmission characteristics of surface waves, Song et al. [14] extracted the initial 100 µs length of measured signals such that the centerline of the extracted signals was positioned at the first negative peak (Figure 3a). On the other hand, Kee and Zhu [15] extracted the initial signals within three times the wavelength such that the centerline of the extracted signals was positioned at the first positive peak (Figure 3b). The extracted signals were then windowed by the Hann function in the form of a biquadratic sine function (Figure 4) to minimize any possible effect of reflected wave components from the bottom and side faces of the specimen [13]. To improve the frequency resolution, a zero-padding process was adopted; 800 zeros were added. The zero-padded signals were transformed from the time domain to the frequency domain through the fast Fourier transform (FFT) function (Figure 5). 

**Figure 6:**
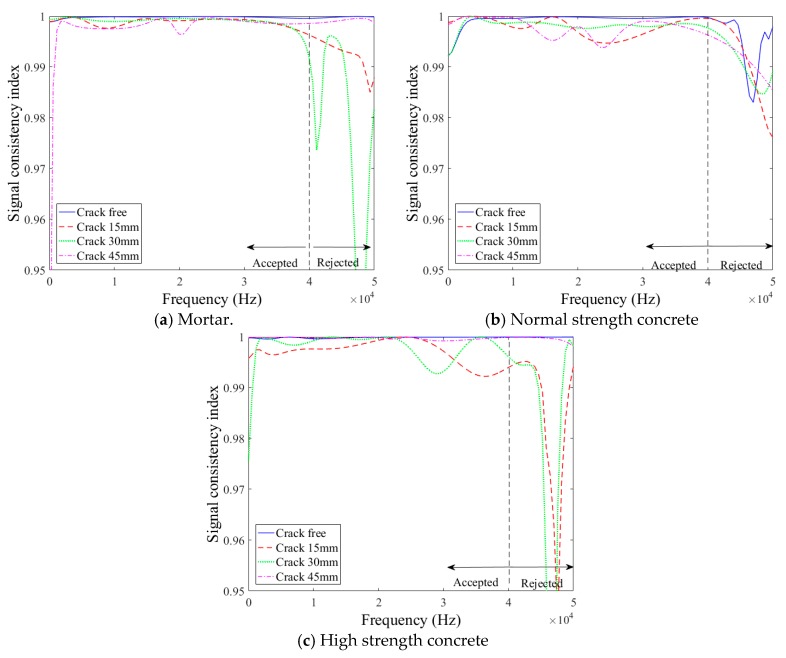
Signal consistency (SC) index data.

In the data processing for the signals of three times the wavelength, there was an unexpected decrease of the signal amplitude near the frequency of 10~15 kHz in the frequency domain, as shown in Figure 5b. In addition, higher signal consistencies in wider frequency domains were observed for the initial 100 µs length of surface wave signals, as shown in Figure 6. Therefore, of the two forms of surface-wave data, the present study used the initial 100 µs length of measured signals per Song et al. [14] for the analysis of the transmission of surface waves. 

Finally, the surface-wave transmission ratio, $T_{BC}\left(f\right)$, between points B and C at a given frequency, is computed using Equation (5), in which $X_{AC}\left(f\right)$ is taken equal to the amplitude of the frequency-domain signal after the crack (plotted by the red hidden line in Figure 5a, for example), and $X_{AB}\left(f\right)$ is taken equal to the amplitude of the frequency-domain signal before the crack (plotted by the blue solid line in Figure 5a for example). 

## 5. Experimental Results

A reliable frequency range for the surface-wave measurements was determined based on the signal consistency (SC) index computed using Equation (6). The frequency domain data had a spectral line spacing (frequency resolution) of 588 Hz. The calculated SC index values are shown in Figure 6. It was found that the SC index drastically decreased above 40 kHz and below 5 kHz for all three mixture types. The surface-wave data within a range of 5 to 40 kHz were consistently above 0.99 and thus considered reliable for use for surface-wave analyses [13,14]. The use of this wide frequency range of surface wave components is needed in the assessment of a wide range of crack depth covering both shallow and deep cracks. 

### 5.1. Effects of Depth

**Figure 7:**
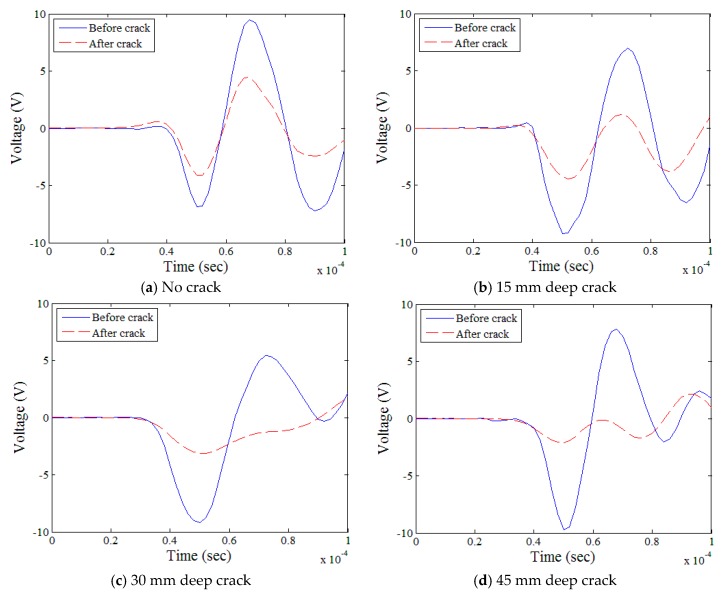
Time-domain surface-wave signals for mortar.

The effects of the crack depth on the obtained time domain signals for the mortar specimens are shown in Figure 7 as an example; similar behaviors were observed in all three mixture types. In general, when the crack depth increased from zero (Figure 7a) to 45 mm (Figure 7d), the peak signal amplitude measured after the crack decreased steadily while that measured before the crack was relatively constant. The potential disruption caused by any reflected waves from the bottom of the specimen was minimized by the application of the Hanning window to the time-domain data, which attenuated the signals arriving later than the first Rayleigh-wave peaks (Figure 4).

**Figure 8:**
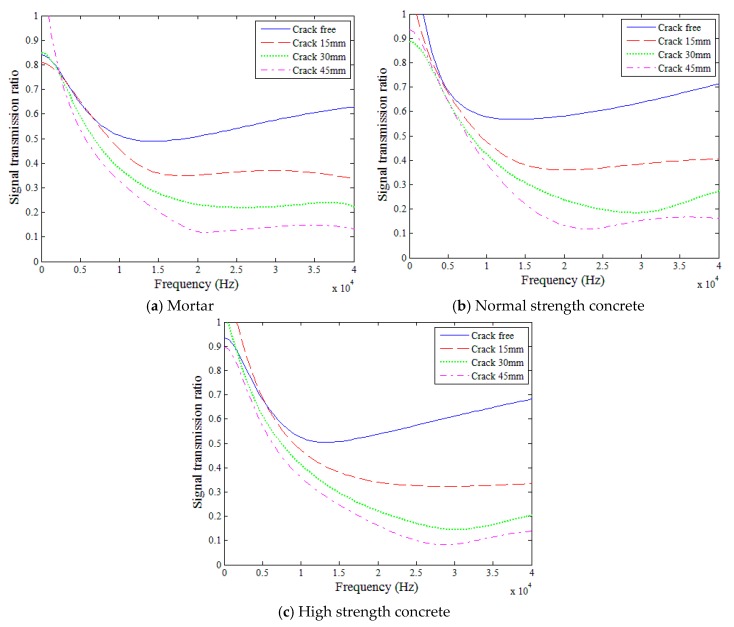
Surface-wave transmission ratios.

Figure 8 compares the surface-wave transmission ratios determined using Equation (5) for four crack depths in each of the three mixture types (i.e., mortar, normal strength concrete, and high strength concrete) for the 5 to 40 kHz frequency range. In each figure, blue, red, green, and magenta lines indicate 0-, 15-, 30-, and 45-mm crack depth cases, respectively. To improve the reliability of the test results, the transmission ratio presented in Figure 8 was determined by the geometric averaging of the data measured five times. The geometric average of the transmission ratios more clearly presents fluctuations and inflection points compared with its arithmetic mean. When the crack depth increased, the transmission ratio generally decreased for all three mixture types throughout the measured frequency domain. 

**Figure 9:**
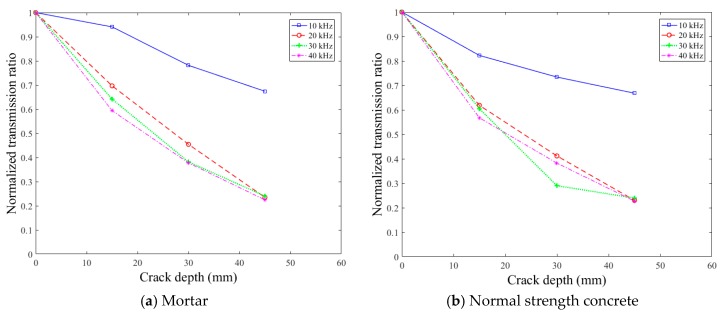

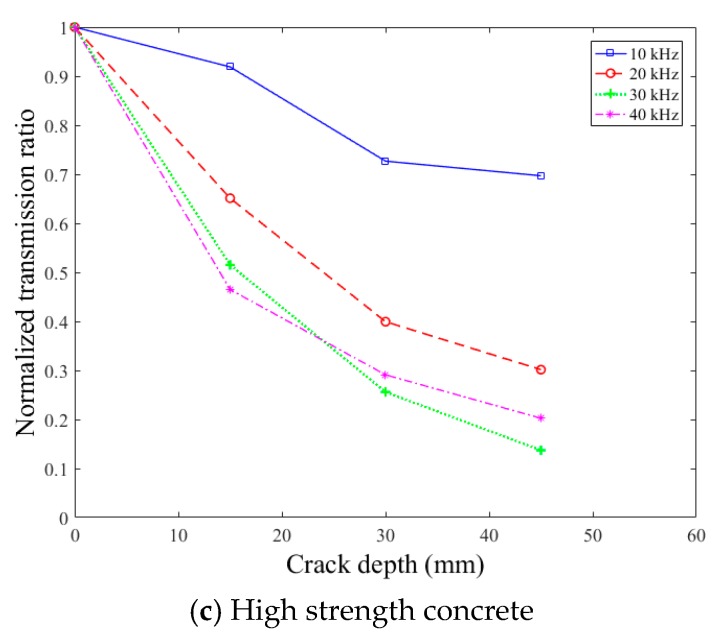
Normalized surface-wave transmission ratios.

Figure 9 presents the surface-wave transmission ratios of 15-, 30-, and 45-mm crack depth cases normalized by the transmission ratio of the crack-free case. The normalized transmission ratios were extracted within the viable frequency range at frequencies of 10, 20, 30, and 40 kHz. With increasing crack depth, the normalized transmission ratio always decreased at all four frequencies in all three mixtures. However, the normalized transmission ratios at 20, 30, and 40 kHz exhibited considerable discrepancies, although they showed relatively similar decreasing trends. Furthermore, the descending trend of the normalized transmission ratio at 10 kHz was quite different from those at 20, 30, and 40 kHz in all three mixtures. 

**Figure 10:**
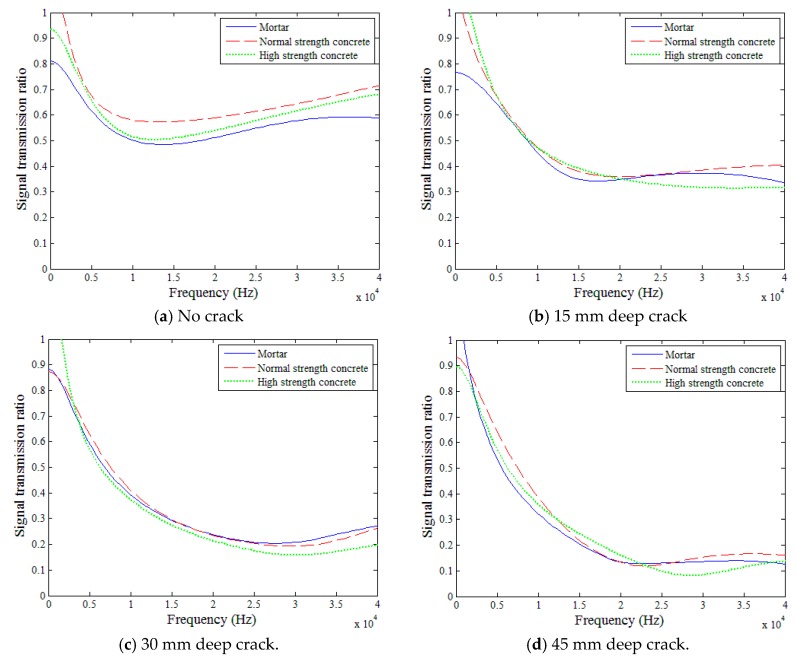
Time-domain surface-wave signals for mortar.

From the results of Figure 9 and Figure 10, it is clear that the surface-wave transmission ratio is highly dependent on the frequency of the waves. The effects of the wave frequency on the normalized transmission ratio are further discussed in Section 5.3 based on the theoretical solutions of Angel and Achenbach [36].

### 5.2. Effects of Mixture Type

Figure 10 compares the surface-wave transmission ratios in the three mixture types for each of the four crack depths (i.e., 0, 15, 30, and 45 mm), for 5 to 40 kHz. In each figure, blue, red, and green lines indicate the cases for mortar, normal strength concrete, and high strength concrete, respectively. The transmission ratios in the three mixtures were quite similar in each of the four crack depth cases throughout the reliable frequency domain (i.e., 5 to 40 kHz). Neither the presence of coarse aggregate nor the strength of concrete appeared to impact the transmission of surface waves across various crack depths. Therefore, it is deemed that the mix proportions (i.e., properties) of concrete had insignificant influence on the surface-wave transmission ratio. This is likely because the wavelengths of the surface waves were much longer than the maximum aggregate size, and the moduli of elasticity of the three mixtures were not much different in the perspective of the surface wave propagation. 

### 5.3. Normalized Transmission Ratio Versus Normalized Crack Depth

**Figure 11:**
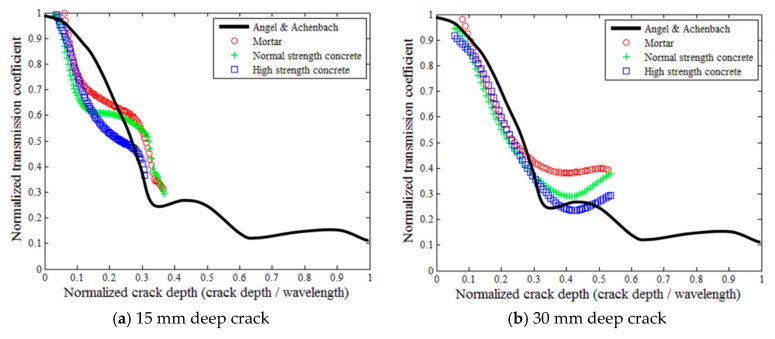

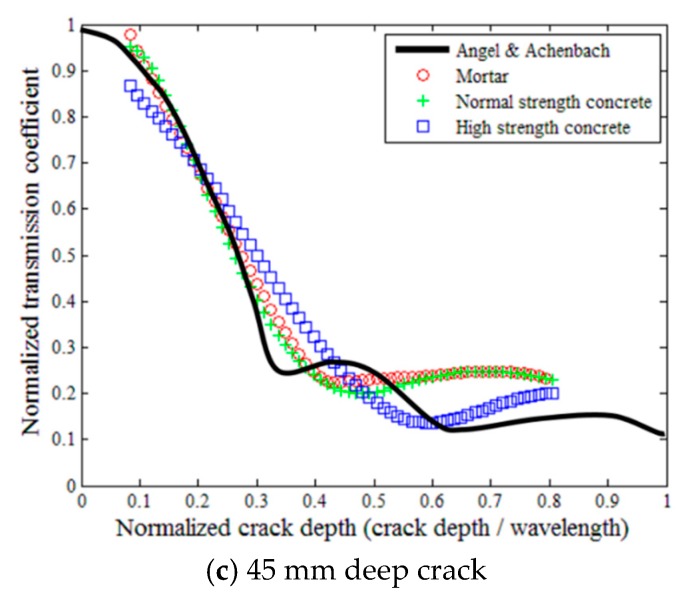
Normalized surface-wave transmission ratios.

Figure 11 plots the normalized transmission ratio as a function of the normalized crack depth for all three mixture types. The normalized crack depth is defined as the crack depth divided by the wavelength. The experimental results are compared with theoretical solutions by Angel and Achenbach [36] to examine the applicability of the theoretical solutions on the estimation of crack depth in concrete. Angel and Achenbach [36] proposed the theoretical solutions for isotropic, linear elastic, and homogeneous materials based on numerical analyses. The red, green, and blue data points represent the experimental results for mortar, normal strength concrete, and high strength concrete, respectively, and the black line represents the theoretical solutions. To ensure the reliability of the analysis, the frequency domain from 5 to 40 kHz in which the SC index was higher than 0.99 (Figure 6) was considered only. To calculate the wavelength, the surface-wave velocity is assumed 2200 m/s, which is commonly used for concrete. Accordingly, the reliable normalized crack depth is limited to 0.27, 0.54, and 0.81 in the cases of 15-, 30-, and 45-mm crack depths, respectively. 

The experimental results of the normalized transmission ratio versus the normalized crack depth presented similar trends to the theoretical solutions, but the differences between the experimental and theoretical results were generally considerable (Figure 11). When the normalized crack depth was less than 0.3, the test results for the 30- and 45-mm crack depth cases were in a good compliance with the theoretical solutions; some previous studies had similar findings [14,15]. In contrast, the 15-mm crack depth cases showed large differences from the theoretical solutions. 

From the above discussions, the relationship between the normalized transmission ratio and the normalized crack depth seems inadequate for the purpose of the estimation of concrete crack depth. Kee and Zhu [21] have noted that relatively small receiver to crack spacings may disrupt surface wave transmission measurements because of nearfield effects, especially for the case of shallow cracks (e.g., 15-mm deep). However, our results reveal relatively little nearfield disruption, as indicated by the good agreement between the experiment and theory, especially at larger crack depths.

### 5.4. Sensitivity of Surface-Wave Spectral Energy to Crack Depth

Although the surface-wave transmission ratio is sensitive to crack depth, it is also dependent on the frequency of waves (Figure 8, Figure 9, Figure 10 and Figure 11) and, moreover, the dependency on the wave frequency displays no clear and consistent trend. Thus, the surface-wave transmission ratio itself appears inappropriate for the estimation of crack depth in concrete. On this viewpoint, the interpretation of surface-wave signal transmission through spectral energy analysis may be more dependable for the purpose of crack depth estimation [16,17]. In this study, a reasonably accurate model for the estimation of crack depth is determined as a function of spectral energy transmission ratio based on the surface-wave measurements obtained considering the effects of concrete mix proportions (i.e., maximum aggregate size, concrete strength). 

**Figure 12:**
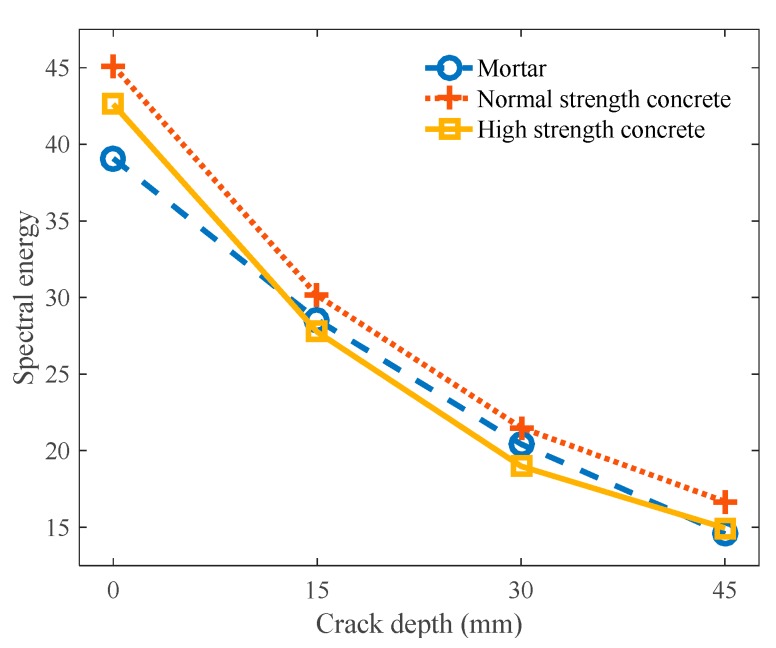
Spectral energy with respect to crack depth.

Figure 12 shows the surface-wave spectral energy with respect to the crack depth in all twelve specimens, calculated by Equation (7); each point corresponds to the average of twenty-five surface-wave measurements conducted on each specimen. The lower and upper frequency limits for the integration of surface-wave transmission ratios in Equation (7) were taken as 5 and 40 kHz, respectively. In general, the measured spectral energy had a good correlation with the crack depth for all three mixtures; the spectral energy decreased in a monotonic fashion as the crack depth increased. The normal strength concrete specimens constantly displayed larger spectral energies than the high-strength concrete specimens as well as the mortar specimens, for all the crack depths. It should be noted, however, that the surface-wave transmission ratio, and thus the spectral energy, are dependent on the testing configuration, specifically the distance between the impact source, sensors, and crack [21]. To resolve this issue, the surface-wave spectral energy transmission ratio defined by Equation (8) can be used as a normalized index [16,17]. 

The repeatability of the measurement of the surface-wave spectral energy transmission ratio for the purpose of crack depth estimation is examined by typical statistical analysis. The spectral energy (SE) for each specimen (having a given mixture type and crack depth) was measured twenty-five times, and the spectral energy transmission ratio (SETR) for each cracked specimen was determined by dividing the SE value for the cracked specimen by the average of twenty-five SE values for the uncracked specimen of the same mixture type. Table 3 summarizes the average, standard deviation, and coefficient of variation (COV) of twenty-five SETR values determined for each specimen. In general, the variation of the SETR values increased as the crack depth increased; the specimen with a 15 mm-depth crack showed the smallest COV regardless of the mixture type, although it had the largest standard deviation in all mixture types. On the other hand, the normal strength concrete specimens exhibited both the smallest standard deviation and COV in all crack depths. Above all, the COV of the SETR values was reasonably small (less than 5.25%) in all the specimens with different mixture types and crack depths. Therefore, it is deemed that the SETR measurement of surface waves is repeatable with a reasonably small variability.

### 5.5. Crack Depth Estimation Model Based on Spectral Energy Transmission Ratio

**Figure 13:**
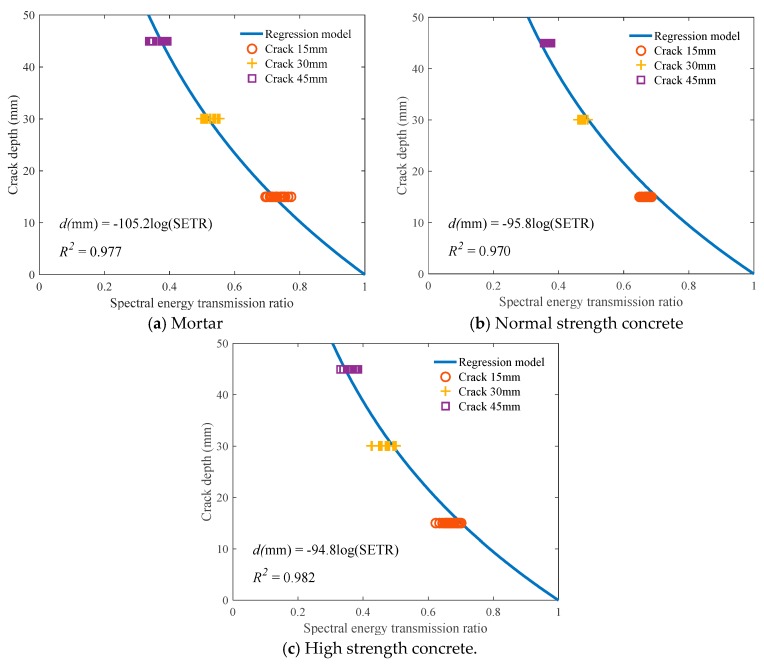
Crack depth as a function of spectral energy transmission ratio.

Figure 13 presents the crack depth as a function of the surface-wave spectral energy transmission ratio (SETR) for the three mixture types; for each mixture type, a regression curve best fit with the data is also illustrated. The experimental data suggest a semi-log correlation between the SETR and crack depth that is consistent among the three mixture types. To ensure statistical confidence for the regression analysis, twenty-five sets of surface wave signals were collected for each specimen. 

The regression curves for the spectral energy transmission ratio (SETR) versus the crack depth in Figure 13 are determined with the x-intercept taken equal to 1, because the SETR is equal to 1 for the case of zero crack depth (i.e., no crack). The one-parameter regression method is used. As a result, the coefficients of determination (*R*^2^) of the logarithmic regression curves for all the three mixture types are higher than 0.97 (Figure 13). Furthermore, the regression functions for the three mixture types are relatively close to each other: d=−105log(SETR) for mortar, d=−95.8log(SETR) for normal strength concrete, and d=−94.8log(SETR) for high strength concrete. This implies that the effects of concrete properties (i.e., maximum aggregate size, concrete strength) on the SETR of surface waves are negligible with the use of a self-calibrating test configuration.

Finally, a surface-wave based model for the estimation of crack depth in concrete is proposed based on the experimental SETR data, ignoring the effects of concrete mix proportions. When all the SETR data for the three mixture types are plotted together, the best fit correlation between the surface-wave SETR and crack depth *d* (mm) is

(9) d=−98.2log(SETR)

The coefficient of determination (*R*^2^) of this regression model is approximately 0.95. It should be noted that this simple single-variable model is capable of taking into account a wide frequency range of waves, and is independent of the mix proportions of concrete.

The accuracy of the proposed spectral-energy based model in Equation (9) is validated using the root mean square error (RMSE) analysis for the twenty-five sets of surface-wave data collected from each specimen. The RMSE divided by the real crack depth in each specimen (RMSE/*d_real_* in Table 4) is also considered for this validation. Table 4 summarizes the RMSE and average error data for all the tested cases; i.e., three mixture types and three crack depths. The RMSE values for all the cases are less than approximately 4.0 mm, and the RMSE/*d_real_* values are less than roughly 13.5%. This indicates that the proposed model will be reasonably accurate to evaluate crack depth in concrete, and spectral energy transmission ratios will be practically consistent in independent surface-wave measurements. However, the shape of the developed SETR curve suggests an upper limit of crack depth that can be reliably measured, considering the steepness of the curve and the natural variability of the SETR data. This condition occurs when the SETR is less than approximately 0.3 (Figure 13), thus the proposed model may be limited to an upper bound of approximately 55~60 mm. With regard to the RMSE/*d_real_* values, the specimens of a 15-mm deep crack exhibit the largest variations among the different crack depth cases regardless of the mixture type.

Finally, when all the mixture cases are considered together (shown in the last column of Table 4), the RMSE values still remain less than approximately 2.8 mm, and the RMSE/*d_real_* values are less than approximately 15.5%. Similarly, the average error of the estimated crack depths by Equation (9) is less than about 2.4 mm. Therefore, it is concluded that when this testing configuration is used, the proposed spectral-energy based model and associated techniques have a promising potential toward practical applications for the assessment of crack depth in concrete structures. Additionally, further studies are needed to examine the applicability of the proposed model on real cracks developed under various loading conditions. 

## 6. Summary and Conclusions

In this study, nondestructive surface wave tests were conducted on lab-scale concrete specimens with varying mix proportions and discontinuity (i.e., crack) depths to better understand the effects of concrete properties and crack depth on surface-wave transmission, and, finally, to develop an accurate and practical model for the estimation of crack depth in concrete. The conclusions presented here are based on the test results obtained from simulated cracks (i.e., notches) with well-defined tips and clear opening faces along the depth. Future research efforts will explore more practical conditions involving real cracks of various configurations (e.g., inclined cracks) and varying test configurations (e.g., distances between impact, sensor, and crack). The findings and conclusions of this study are summarized as follows:

(1) The surface-wave transmission ratio constantly decreased as the crack depth increased, for all the different mixture types throughout the measured frequency domain. However, the decreasing rate of the surface-wave transmission ratio was dependent on the wave frequency.
(2) Both the presence of coarse aggregate and the strength of concrete had negligible influences on the propagation and transmission of surface waves across simulated cracks of various depths in concrete. This confirms the surface-wave technique combined with the self-calibrating test scheme for applications to concrete structures made of a variety of mix proportions.
(3) The relationship between the normalized transmission ratio and normalized crack depth obtained from the measured surface-wave data showed similar trends to the theoretical solutions proposed by Angel and Achenbach [36]. However, the discrepancies between the experimental and theoretical results were not negligible, especially for the smallest crack depth measured.
(4) The measured surface-wave spectral energy had a strong inverse correlation with the crack depth for all the different mixtures. The variation of the spectral energy transmission ratio (SETR) was reasonably small in all the specimens with different mixture types and crack depths, so that the SETR measurement of surface waves is deemed to be reliable with high statistical confidence.
(5) A model for the estimation of crack depth in concrete is proposed based on the measured surface-wave SETR data, which assumes a semi-log correlation between the SETR and crack depth. The coefficient of determination of the proposed model was approximately 0.95, and the RMSE values were relatively small, compared with the test data. This simple single-variable model is capable of taking into account a wide frequency range of waves, and is independent of the mix proportions of concrete, but has a likely upper limit of crack depth of 55 to 60mm.

## Figures and Tables

**Table 1:** Crack depths and mix proportions of the test specimens.

Specimen Label	Mixture Type	Crack Depth (mm)	Mix Proportions (kg/m^3^)				
			Cement	Water	Fine Aggregate	Coarse Aggregate	Super-Plasticizer
M0	Mortar	0	578 (1)	260 (0.45)	1445 (2.5)	-	2.31 (0.004)
M15		15					
M30		30					
M45		45					
NC0	Normal strength concrete	0	374 (1)	168 (0.45)	935 (2.5)	935 (2.5)	1.50 (0.004)
NC15		15					
NC30		30					
NC45		45					
HC0	High strength concrete	0	434 (1)	152 (0.35)	932 (2.15)	932 (2.15)	3.46 (0.008)
HC15		15					
HC30		30					
HC45		45					

Note: The number in parentheses indicates the mass of the material normalized with regard to that of cement.

**Table 2:** Compression test results of the three mixtures.

Mixture Type	Compressive Strength (MPa)		Strain at Compressive Strength		Modulus of Elasticity, *E_c_* (GPa)	
	Average	Standard Deviation	Average	Standard Deviation	Average	Standard Deviation
Mortar	39.5	2.05	5.73 × 10^−3^	1.74 × 10^−3^	21.6	2.30
Normal strength concrete	40.1	1.64	2.07 × 10^−3^	0.33 × 10^−3^	30.9	1.19
High strength concrete	57.4	1.67	2.51 × 10^−3^	0.19 × 10^−3^	33.1	1.24

**Table 3:** Spectral energy transmission ratios (SETRs) for all twelve specimens.

Mixture Type		Mortar			Normal Strength Concrete			High Strength Concrete		
Crack Depth (mm)		15	30	45	15	30	45	15	30	45
SETR (%)	Average	73.2	52.3	37.1	66.6	47.3	36.3	67.1	46.5	36.6
	Standard deviation	2.15	1.81	1.95	0.94	0.76	0.72	2.02	1.99	1.41
	Coefficient of variation	2.93	3.46	5.25	1.41	1.61	1.99	3.01	4.28	3.85

**Table 4:** Root mean square error (RMSE) analysis of the proposed model in Equation (9) for measured SETRs.

Mixture Type	Mortar			Normal Strength Concrete			High Strength Concrete			All Mix Cases Together		
Crack Depth, *d_real_* (mm)	15	30	45	15	30	45	15	30	45	15	30	45
RMSE (mm)	1.54	1.61	2.48	2.02	1.37	2.94	1.91	2.33	3.94	2.32	2.72	2.76
RMSE/*d_real_* (%)	10.3	5.4	5.5	13.5	4.6	6.5	12.7	7.8	8.8	15.5	9.1	6.1
Average error (mm)	1.31	1.41	2.10	1.93	1.23	2.82	1.51	1.91	3.63	2.06	2.33	2.36
